# Highlights of the Virtual Society for Cardiovascular Magnetic Resonance 2022 Scientific Conference: CMR: improving cardiovascular care around the world

**DOI:** 10.1186/s12968-022-00870-4

**Published:** 2022-06-20

**Authors:** Vineeta Ojha, Omar K. Khalique, Rishabh Khurana, Daniel Lorenzatti, Steve W. Leung, Benny Lawton, Timothy C. Slesnick, Joao C. Cavalcante, Chiara-Bucciarelli Ducci, Amit R. Patel, Claudia C. Prieto, Sven Plein, Subha V. Raman, Michael Salerno, Purvi Parwani

**Affiliations:** 1 All India Institute of Medical Sciences, New Delhi, India; 2 Saint Francis Hospital and Catholic Health, Roslyn, NY USA; 3 Hospital Universitari de Vic, Vic, Barcelona, Spain; 4 Gill Heart and Vascular Institute, University of Kentucky, Lexington, KY USA; 5 St Joseph’s Hospital, Malpas, Newport, Wales; 6 Emory University School of Medicine, Atlanta, GA USA; 7 Minneapolis Heart Institute, Minneapolis, MN USA; 8 Royal Brompton and Harefield Hospitals, King’s College London, London, UK; 9 Division of Cardiovascular Medicine, University of Virginia, Charlottesville, VA USA; 10 School of Biomedical Engineering and Imaging Sciences, King’s College London, London, UK; 11 Leeds Institute of Cardiovascular and Metabolic Medicine, University of Leeds, Leeds, UK; 12 Indiana University Cardiovascular Institute and Krannert Cardiovascular Research Center, Indianapolis, IN USA; 13 Department of Medicine, Stanford University, Stanford, CA USA; 14 Division of Cardiology, Department of Medicine, Loma Linda University Health, Loma Linda University Medical Center, Loma Linda, CA USA

**Keywords:** Cardiovascular magnetic resonance, Valvular heart disease, Non-ischemic cardiomyopathy, Rapid cardiac magnetic resonance, Artificial intelligence, 4D flow, Congenital heart disease, 3D printing, Early career

## Abstract

The 25th Society for Cardiovascular Magnetic Resonance (SCMR) Annual Scientific Sessions saw 1524 registered participants from more than 50 countries attending the meeting virtually. Supporting the theme “CMR: Improving Cardiovascular Care Around the World”, the meeting included 179 invited talks, 52 sessions including 3 plenary sessions, 2 keynote talks, and a total of 93 cases and 416 posters. The sessions were designed so as to showcase the multifaceted role of cardiovascular magnetic resonance (CMR) in identifying and prognosticating various myocardial pathologies. Additionally, various social networking sessions as well as fun activities were organized. The major areas of focus for the future are likely to be rapid efficient and high value CMR exams, automated and quantitative acquisition and post-processing using artificial intelligence and machine learning, multi-contrast imaging and advanced vascular imaging including 4D flow.

## SCMR 2022: “CMR: Improving Cardiovascular Care Around the World”

Cardiovascular magnetic resonance (CMR) is an integral part of cardiovascular medicine. With the unprecedented advances in CMR, this field has attracted the rapidly growing interest of the clinical and scientific communities throughout the world [1]. The Society for Cardiovascular Magnetic Resonance (SCMR) Annual Scientific Sessions is the largest worldwide annual gathering of physicians, scientists, and others with an interest in CMR and in 2022 celebrated its 25th iteration. Initially planned as a hybrid meeting comprising both in-person and virtual modes, the meeting had to be switched to an entirely virtual platform with less than 2 months’ notice. Despite this late change, the SCMR 25th Annual Scientific Sessions, presented virtually from February 2–5, 2022, were immensely successful with 1524 registered participants from over 50 countries and 6 continents attending.

In addition to the main meeting, three large pre-conferences were held—the Physicians and Peds/Congenital Heart Disease (CHD) preconference and the SCMR/International Society of Magnetic Resonance in Medicine (ISMRM) workshop. The meeting organization was led by Dr. Michael Salerno and a balance of cardiologists, radiologists, scientists, technologists, and others comprising the SCMR Program Committee. Special effort was made to ensure that the faculty represented a balance of male and female radiologists, cardiologists, scientists, and allied health professionals.

**Fig. 1:**
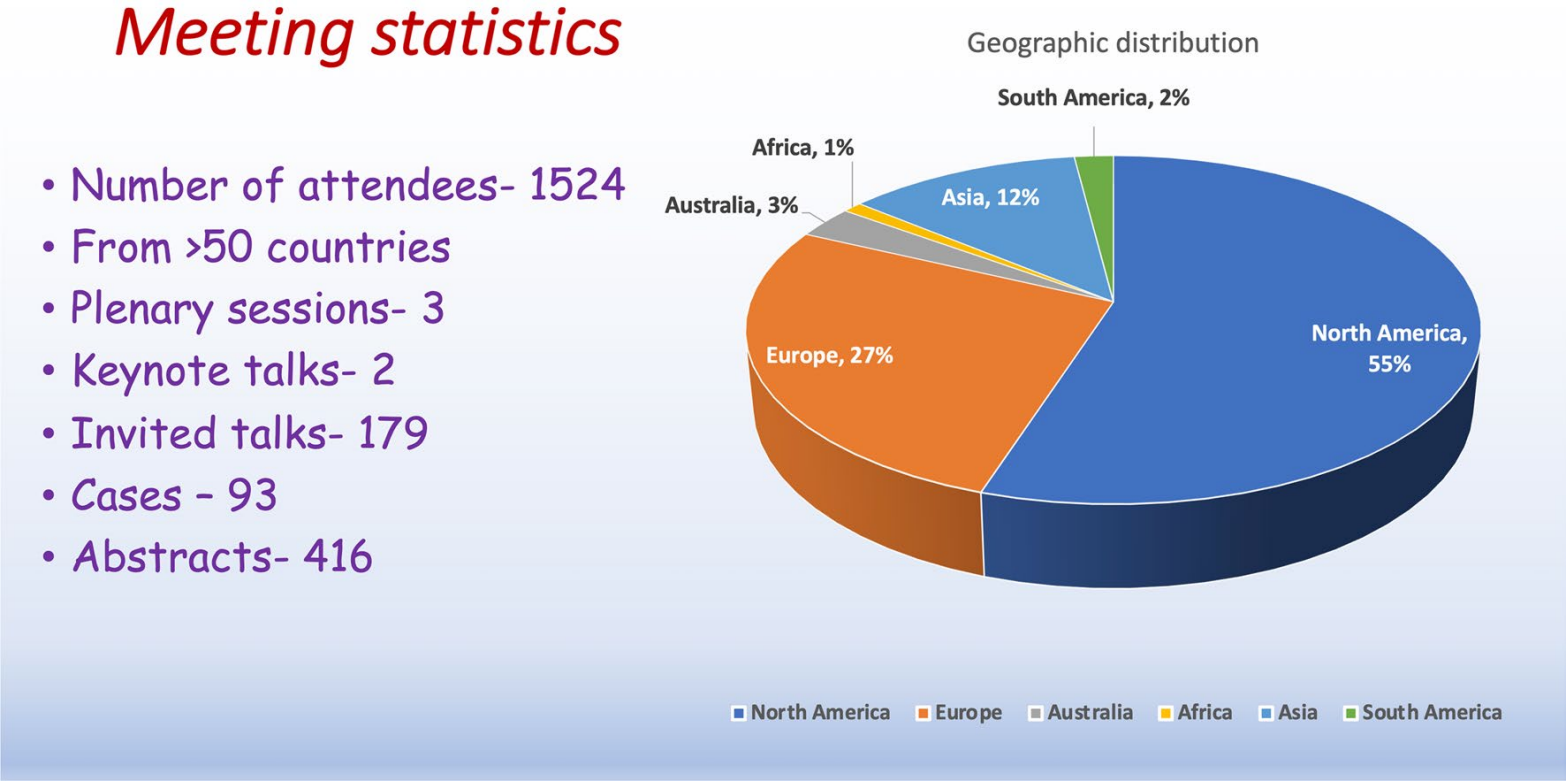
Statistics of the Society for Cardiovascular Magnetic Resonance (SCMR) 2022 Annual Scientific Sessions meeting. The pie chart shows the distribution of participants across the continents

Supporting the theme “CMR: Improving Cardiovascular Care Around the World”, the meeting included 179 invited talks, 52 sessions including 3 plenary sessions, 2 keynote talks, and a total of 93 cases and 416 posters (Fig. 1). The opening plenary session “The past, present and the future of SCMR” recorded the highest attendance. The second plenary, “CMR: Delivering Value”, discussed value from the perspective of different stakeholders including physicians, hospital administrators, and a patient who underwent CMR as part of her care. In this session, the American College of Cardiology (ACC) president Dr. Dipti Itchhaporia also shared her views on how CMR can offer value to various stake holders. For the 3rd Plenary, “Towards the 10-min CMR Exam”, various approaches including real time, 3D all-in-one, and artificial intelligence (AI) for rapid imaging were discussed. Dr. Harlan Krumholz presented the keynote lecture where he discussed how CMR plays a pivotal role in the era of precision medicine. The two outstanding Gold Medal lectures (2021 recipients) for this year were delivered by Drs. Matthias Stuber and Matthias Friedrich. Dr. Stuber shared his exciting and inspiring journey in this field and Dr. Friedrich shared his vision for the future of CMR.

In this present manuscript we summarize the highlights from SCMR 2022, organized by different cardiovascular pathologies and CMR techniques.

## Non-ischemic cardiomyopathy

The established utility of CMR in the diagnosis and management of non-ischemic cardiomyopathy (NICM) was well demonstrated in the variety of sessions focussing mainly upon case-based discussion of the applications of CMR. The talks highlighted the expanding role of CMR in assessing myocardial structure and function, wall motion abnormalities, myocardial mechanics, perfusion, viability, valvular function, advanced myocardial tissue characterization, risk stratification and prognostication of patients with NICM [2]. The session “case-based approach to NICM” comprised of cases highlighting the unique role of CMR in diagnosing cardiac amyloidosis. We also saw cases highlighting the difference between “excess LV trabeculation (imaging diagnosis)” vs “non-compaction (phenotype)” as well as sarcomere mutation positive (more late gadolinium enhancement (LGE) and left ventricular (LV) outflow obstruction) vs mutation negative (less LGE and LV outflow obstruction) hypertrophic cardiomyopathies [3, 4]. A couple cases of desmoplakin associated arrhythmogenic cardiomyopathy were presented at the meeting, highlighting the utility of CMR in diagnosing this recently described entity [5]. The session on CMR reading with the expert was one-of a kind with Early Career members presenting cases followed by a robust discussion by the senior CMR experts. In another session on cases in NICM, the speakers took us through various rare presentations like rheumatoid arthritis manifesting as acute myocarditis and pericarditis as well as a case of cardiac amyloidosis complicated by microvascular disease diagnosed by quantitative myocardial perfusion. Additionally, the delegates learned how native T1 mapping can help in diagnosing various stages of Anderson-Fabry disease—reduced T1 times in the initial accumulation phase, reduced T1 and elevated T2 in the inflammatory phase and pseudo-normalisation of T1 in the fibrosis stage [6]. A session dedicated to myocarditis had an esteemed panel discussing the new Lake-Louise criteria for diagnosing myocarditis as well as sharing their experience of how CMR helps in detecting Coronavirus disease 2019 (COVID-19) illness and COVID-19 vaccine related myocardial injury in normal population as well as athletes [7].

## Valvular heart disease

The session on valvular heart disease (VHD) focused on the fundamentals, and provided tips and tricks on VHD assessment by CMR by luminary Dr. Dipan Shah. Substantial focus was given to 4D flow imaging particularly in bicuspid valve patients [8]. Dr. Andrea Guala demonstrated that co-localized 4D flow wall shear stress predicts future aortopathy in bicuspid aortic valve patients. Once severe aortic stenosis (AS) sets in, the aortic hemodynamics or stiffness may not be different between bicuspid and trileaflet AS patients. Innovation at Northwestern University was highlighted, demonstrating enhanced assessment of 4D flow intra-aortic segmental flow energetics and vorticity parameters in less than 5 min. In valvular regurgitation topic, a multi-center study led by Dr. Tom Kai Ming Wang elucidated CMR threshold values of regurgitant volume of 35 ml and regurgitant fraction of 29% for aortic regurgitation and regurgitant volume of 33 ml and regurgitant fraction of 34% for mitral regurgitation for progression to surgery. LV remodelling appears to be reduced after aortic valve surgery if the residual aortic regurgitant volume assessed by phase contrastof the sinotubular junction or ascending aorta is >  = 20 ml. Moreover, in the sessions focusing on the understanding of the “forgotten tricuspid valve”, severe tricuspid regurgitation and its impact on the right ventricle (RV) by CMR was highlighted. RV insertion point LGE is associated with a more dilated RV and lower RV ejection fraction in the setting of severe tricuspid regurgitation.

## Early career sessions

Early Career sessions at SCMR 2022 focused on three parallel themes. The organizing committee and Early Career Council not only designed the sessions on career advancement and professional development, but also wanted to give early career professionals the opportunity to network with mentors as well as other early career professionals across the globe. For professional growth sessions, the most popular session was “Early Career Challenges and Growth as A CMR Imager and Researcher—Hearing from Mentor–Mentee Duos”. Five successful mentor–mentee duos shared their journey from the beginning, and discussed how mentorship can be a key for growth of a mentee. “Launch Your CMR Career—Clinical and Research” sessions focused on finding a clinical and research niche as a multimodality imager, tips on key components of a CMR service, choice of the first scanner in new CMR lab, training of the CMR technologist, collaboration with industry, core lab set up and choice of scanner for early career professionals across the world. Early career professional growth sessions were dedicated on discussion on developing leadership skillsets through different stages of one’s career, discussed tips for successful radiology-cardiology collaboration for clinical growth and need for a coach, mentor and sponsors for the academic growth of early career CMR imager. Last but not the least, the early career professional challenges across the world gave us a perspective on common challenges early career professionals face in the field of CMR.

## Rapid CMR protocols

Another highly attended session at SCMR 2022 was the Friday plenary session on rapid CMR “Towards the 10 min CMR Exam”. The session discussed some of the technical advances that should allow us to further reduce the CMR examination time without compromising image and diagnostic quality. Recently, CMR protocols of 30 min or shorter duration with routine techniques have been described in a SCMR white paper [9]. Here, further technical developments in the areas of real time imaging, 3D whole-heart multiparametric and multicontrast imaging and AI-empowered solutions to reduce scan and analysis times were discussed to enable highly efficient, comprehensive, and easy to perform CMR exams that may enable increased access to CMR worldwide in the future. These new approaches bring new associated challenges such as 3D quantification, analysis and interpretation for whole-heart techniques, validation and domain shift (due to differences across scanners and sites) for AI solutions, as well as the importance of common efforts on reproducibility and quality assurance, which were enthusiastically discussed during this session. The panel also discussed the opportunity for non-contrast enhanced techniques as an important direction that may also help to simplify exams and reduce scan times. Lastly, a large number of abstracts presented during SCMR 2022 focused on this topic and gave us a promising perspective on moving towards the 10-min CMR exam. We hope to see these developments move towards clinical translation next year at SCMR 2023.

## Pediatric CMR and congenital heart disease

The pediatric and congenital heart disease (CHD) sessions at SCMR 2022 provided attendees with a wide range of offerings: from building blocks to launch a junior career all the way to state-of-the-art talks on cutting edge research. The Pediatric/CHD Pre-conference spanned seven sessions, including use of CMR in patients with implanted cardiac devices, the expanding role of CMR in patients with pulmonary hypertension, and a discussion on the added challenges of 3 T CMR in pediatric patients [10, 11]. Friday afternoon brought a comprehensive session focusing on the emerging role of surgical planning using CMR imaging, with examples including 3D printing, “flat screen” visualization, as well as the role of virtual reality [12]. Important to this discussion was the perspective of a pediatric cardiac surgeon to drive home the aspects of this work that most influence his direct patient care. The other highlighted session was a lively discussion of the future direction of our field, and in particular whether more comprehensive data is paramount or faster scanning techniques are key. This session featured two excellent talks, but just as important had a full 30 min for discussion. Not surprisingly, both speakers agreed that in an ideal world we will have both increased speed and more comprehensive data. Faster sequences, reconstructions powered by convolutional neural networks (CNN)’s and AI, dedicated pediatric coils, etc. will allow us to go faster, and with our increased scan efficiency we be able to generate more comprehensive data, including 3D cine, stress CMR with techniques like oxygen sensitive imaging, and myocardial energetics including use of ^31^P CMR spectroscopy [13, 14]. Interleaved with these didactic sessions were a host of exciting original research abstracts. Two themes which emerged centered around sequence innovation and virtual surgical planning using segmentation and computational fluid dynamic techniques. Finally, the 3D + special interest group hosted a series of lectures describing both historical perspectives on the techniques and allowing a peek into the future of this space.

*Women’s cardiovascular* One of the highlights of SCMR 2022 was focus on quantitative perfusion for evaluation of Ischemia and No Obstructive Coronary Artery disease (INOCA). During the session on women’s cardiovascular disease, Dr. Chiara Bucciarelli-Ducci spoke at length about use of quantitative CMR perfusion for diagnosis of microvascular disease (MVD), and how an automated pixel-wise perfusion mapping technique on CMR can be used to detect physiologically significant coronary artery disease (CAD), defined by fractional flow reserve, MVD defined by index of microcirculatory resistance ≥ 25, and to differentiate MVD from multivessel coronary disease [15]. Stress myocardial blood flow (MBF) < 2.23 ml/gm/min is accurate for detection of MVD in the absence of obstructive CAD. Additionally, in patients with known or suspected CAD, reduced stress MBF and myocardial perfusion reserve (MPR, the ratio of stress to rest MBF) measured automatically using CMR perfusion mapping provides a strong, independent predictor of adverse cardiovascular outcomes [16].

## CMR in cardio-oncology

CMR in cardio-oncology session highlighted the importance of CMR strain as a tool in early detection of cardiotoxicity, prediction of its prognosis and the role of CMR strain in guiding therapy & management. The need for more literature to correlate different CMR strain techniques and the need to include CMR strain parameters in cardio-oncology trials was emphasized to understand impact of left ventricular (LV) strain on diagnosis and management. The multimodality guidelines for imaging cardiotoxicity, emphasizing the need for early initiation of heart failure therapy leading to better outcomes were discussed at length in these sessions [17]. SCMR Cardio-oncology special interest group meeting discussed the recent updates and future goals for promoting education and enhancing the field of cardio-oncology by increasing use of CMR. The “How I do CMR” for cardio-oncology PowerPoint is accessible on the SCMR website as a useful tool for standardisation of protocols in various institutions across the globe. Furthermore, the potential role of CMR in pediatric cardio-oncology patients was emphasised.

## Ischemia and coronary artery disease

In late 2021, the highly anticipated new Guidelines for the Evaluation and Diagnosis of Chest Pain was published with endorsement by multiple societies including SCMR [18]. In ischemia and CAD sessions, the four major modalities were discussed by experts in each field. With the benefit of portability, echocardiography has high versatility in the assessment of chest pain at rest to assess for wall motion abnormalities, valvular heart disease, aortic dissection and pericardial disease. With coronary computed tomography (CT) angiography, anatomic assessment of CAD can be made, as well as physiologic flow prediction based on fractional flow reserve-CT in those with 40–90% stenoses. With stress positron emission tomography (PET), there is an added benefit of quantitative myocardial blood flow reserve for detection of microvascular disease. Stress CMR was emphasised to be the most useful not only, in identification of obstructive CAD and scar burden, but also in making a diagnosis in patients with INOCA, myocardial infarction with no obstructive coronary arteries (MINOCA), suspected acute pericarditis/myocarditis and suspected microvascular disease. It was again highlighted that all modalities have their individual strengths and weaknesses and choice of stress modality depends on local expertise and availability in addition to advantages for a given patient.

## Cardiac masses and pericardial diseases

During the physician’s pre-conference course, Dr. Ana Barac, led the delegates through the basics of evaluation of cardiac masses. She highlighted the CMR protocol comprised of ventricular functional analysis, T1 and T2 weighted sequences with and without fat suppression through the mass and surrounding structures, first pass perfusion with slices through the mass lesion, repeat T1 weighted fast spin echo with fat suppression (early after gadolinium-based contrast agent administration), repeat selected balanced steady state free precession cine images post contrast (optional), followed by LGE, including long TI imaging (thrombus vs tumour) and serial imaging (hypoperfused tumour necrotic core vs thrombus) [19]. CMR has a high diagnostic accuracy, and is an excellent independent predictor of long-term mortality, in patients with suspected cardiac tumour [20]. The session was followed by a detailed discussion on pericardial evaluation on CMR and a complete spectrum of pericardial diseases were shown. Very interesting and rare cardiac/pericardiac mass cases were presented, highlighting the importance of clinical evaluation as well as various CMR sequences, hence a multiparametric approach.

## Vascular imaging and 4D flow

4D flow imaging has been the new kid on the block in the #WhyCMR world in the last few years. In this year's program, a complete dedicated session regarding the present and future of the technique was held. As Dr. Joshua Robinson depicted, 4D flow CMR is moving from being a research tool to answering real-life clinical questions, with a central role in congenital heart disease patients but also increasingly used for the direct assessment of valvular heart disease due to the valve tracking feature provided in various processing softwares. One of the pioneers in the field, Dr. Michael Markl, showed the advantages of the application of compressed sensing, AI and deep learning to 4D flow in order to accelerate the acquisition (5 min), reconstruction (30 times faster with AI) and to automatize segmentation [21]. He also presented his group's work on a novel fully self-gated and free running sequence, the so called "5D flow", which provides respiratory and cardiac resolved 3D data with astonishing image quality [22].

The field of CMR coronary imaging has recently advanced thanks to faster and higher spatial resolution sequences. In the aforementioned Friday plenary "Towards a 10 min CMR Exam”, it was shown that comprehensive, non-invasive imaging of coronary atherosclerosis is possible with CMR using a 3D multiparametric sequence in only 10 min [23]. This sequence provides simultaneous 3D whole-heart bright and black blood datasets that allow characterization of luminal stenosis as well as plaque features (intraplaque haemorrhage, thrombus, remodelling).

## Case vignettes, case of the week competition

A primary focus of this year’s meeting was on case-based CMR learning which covered ischemic heart diseases, NICM, cardiac and pericardial masses, CHD and VHD. These cases covered the applications of cine images, mapping, LGE, perfusion imaging, extracellular volume (ECV) quantification and strain imaging in the diagnosis and management of a wide spectrum of both common and rare cardiac pathologies. In addition, there was a competition among the Top 5 Cases of the Week for year 2021 (now “Cases of SCMR”) which included tricuspid valve papillary fibroma, endocardial fibroelastosis in congenital pulmonary stenosis, coronary sinus atrial septal defect by 4D flow CMR, role of CMR post-LV assist device explanation and RV outflow obstruction in a patient with pectus carinatum. In addition to the recognition at Scientific Sessions, cases of SCMR for a particular year are collated will be published in *Journal of Cardiovascular Magnetic Resonance* (JCMR), making this an exciting option to those wanting to submit their interesting CMR cases as brief case reports.

## Advanced methods in CMR

### CMR fingerprinting

Every year, there are exciting advances in the development of new CMR sequences. One of the more prominent topics presented this year was related to the ongoing advances in CMR fingerprinting. This involves the development of multi-dimensional, multi-parametric quantitative CMR sequences, which incorporate different types of tissue characterization/function into a single acquisition. This includes a combination of T1 mapping, T2 mapping, T2*, fat fraction, T1rho, cine imaging in 2D or 3D with perhaps free breathing/self-gated acquisition to improve CMR efficiency [24, 25, 26].

### Artificial intelligence/ machine learning

The hot topic of incorporation of AI into the practice of CMR was discussed in detail at SCMR 2022. Machine learning AI can improve CMR performance with more data over time. AI permeates not only the image reconstruction allowing free breathing ungated image acquisition to produce useful images for clinical assessment, but also post-processing quantification such as cardiac chamber volume calculation and myocardial perfusion. There is now investigation into using machine learning to identify cardiac diseases and provide prognostic information. Radiomics is the ability to extract data from images that is not readily identified by the naked eye, but can be derived from machine learning algorithms. There are ongoing projects which investigate the assessment of cine images with myocardial deformation analysis to identify cardiac pathology, texture analysis of cine images to determine areas of scar without the use of contrast, and the use of cine images to obtain displacement encoding with stimulated echo (DENSE) type strain data [27, 28].

### Interventional CMR

There have been 20 years of development in interventional CMR. Dr. Robert Lederman discussed various barriers have prevented the wide spread use of the technology, and the technologic advances that has occurred. The development of low field (0.55 T) CMR may help with reduced specific absorption rate (SAR) limitations and heating and easier development of CMR safe interventional equipment. The current work has been assessing how to use hemodynamic data with catheterization and CMR volumetric data to create pressure–volume loops to enhance the understanding of pathophysiology of disease states. These concepts were elucidated by the esteemed panel at this year’s meeting.

### Exercise CMR

Dr. Orlando Simonetti reviewed the various methods of performing exercise CMR and the utility of the test. This can include supine bicycles/steppers, hand grip exercise equipment and hydraulic driven treadmills. Exercise CMR can be used to assess a patient’s cardiac function during exercise, and may have particular value in the setting of unexplained dyspnea. Exercise CMR has been used to assess obstructive CAD, dilated cardiomyopathy, and heart failure with preserved ejection fraction. Future directions in this area include the determination of systemic metabolic oxygen consumption with CMR oximetry and real-time flow imaging and post-processing to see stroke volume and cardiac output changes during exercise [29, 30, 31].

## Technologists’ track

As the 25th SCMR annual scientific sessions were presented virtually, the Technologist Track consisted of 18 pre-recorded presentations which formed 6 unique and interesting sessions. All the presentations were available as on demand videos which could be viewed by the delegates at any time during the 4 days of the conference. This made it exceedingly convenient no matter where in the world, and at whatever time the delegates wished to view the videos. The technologist track benefited greatly from the 6 live breakout Zoom sessions where two moderators and the presenters could discuss at length the topics of their pre-recorded videos, and answer questions submitted into the chat box by the live audience. Each session was well attended and discussions on topics such as CMR physics, safety, and pediatric imaging were enjoyed. A particularly interesting discussion took place in the Advances in CMR session, which included the previous President and Gold Member winner Prof Matthias Stuber. The fascinating discussion focused on the evolving role of the technologist in the setting of potential reductions in imaging time and increasing amounts of post processing and data evaluation over the coming years because of ongoing technological innovations in sequence design and the expanding use of AI in image acquisition.

## Other activities

**Fig. 2:**
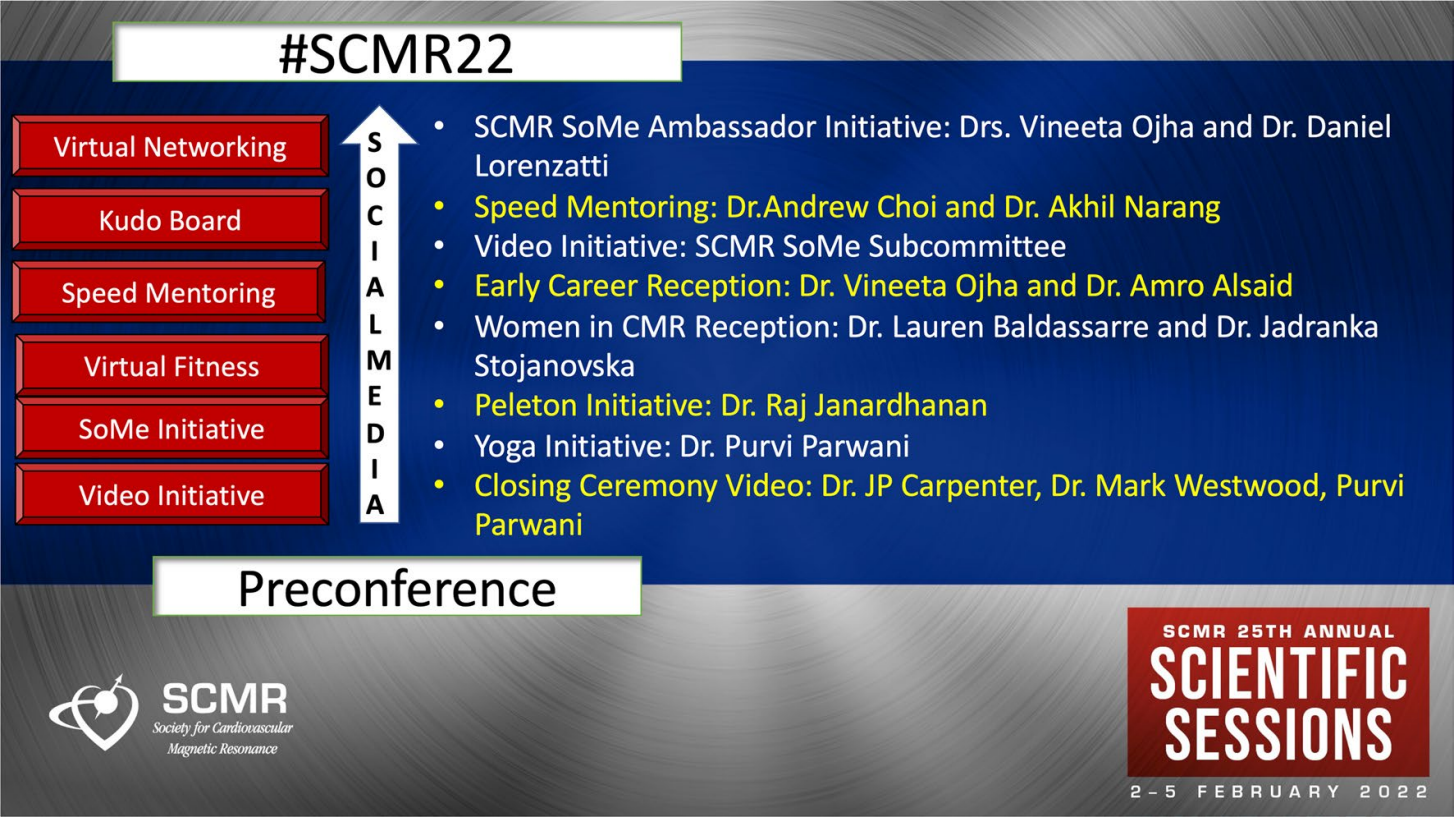
The social media initiative and various events and activities organized during SCMR 2022 Annual Scientific Sessions meeting

The early career reception held on Wednesday evening was a great platform for the exchange of ideas between the early career CMR imagers. More than 70 early career members from more than 15 countries met virtually at this event. The speed mentoring session for the early career and the fellows in training allowed the early career and fellows in training to meet with various CMR leaders. The Women in CMR reception held on Thursday saw the incredible women power of SCMR tuning in to have an interesting discussion on work-life balance, time management, self-care, importance of family and priorities in different spheres of life. Similar to last year, the SCMR video initiative included a few videos released on social media in which an early career member interviewed one member from the SCMR leadership on a particular session or topic of interest. Events like yoga and Peloton ride were necessary virtual getaways from the busy conference schedule and drew many fitness enthusiasts. The SCMR kudo board allowed delegates to scribble their thoughts and previous memories and have a virtual chat with each other. The Gathertown virtual party was a nice alternative to the in-person afterparty of the SCMR. The SCMR quiz conducted by the incredible quizmasters Drs. Mark Westwood, Patricia Bandettini and John-Paul Carpenter registered one of the highest attendances and a complete amalgam of fun and education (Fig. 2).

Many coveted awards were announced at the closing ceremony. There were two deserving winners for the SCMR Seed Grant award—Dr. Justin Canada and team for their study on non-invasive CMR oximetry with exercise stress CMR and cardiopulmonary exercise test and Dr. Junyu Wang and team for “Deep learning-based rapid Spiral Image Reconstruction”. Dr. Warren Manning, *JCMR* Editor-in Chief, presented the *JCMR* awards. The Gerald M. Prohost award for the best scientific manuscript for 2021 went to Dr. Theo Pezel and colleagues for their study on the prognostic value of vasodilator stress perfusion CMRafter inconclusive stress testing [32]. Dr Angelica Romero Daza and colleagues were the Pohost runner’s up for their article on assessment of morpho-functional features of mitral valve prolapse on CMR [33]. The Dudley Pennell award for the manuscript contributing the most to the June 2021 *JCMR* impact factor (currently 5.364) was presented to Dr Wnjia Bai and colleagues for their article on automated CMR image analysis by fully convolutional neural networks [34]. Dr. José Fernando Rodríguez-Palomares and colleagues were the runner’s up for their article on assessment of aortic dilatation by 4D flow in bicuspid aortic valve [35]. The Early Career award winners were Dr. Quiang Zhang for his study on virtual native enhancement (Clinical category), Dr. Soham Shah for his study on role of eplerenone in promoting anti-inflammatory epicardial adipose tissue in mice (Translational category), and Dr. Tianle Cao for his study on non-electrocardiogram-triggered free breathing T1,T2,T2* mapping with CMR multitasking (Basic science category). The Winner of the Case of the Week contest was Dr. Daniel Clark for his case of ventricular tachycardia and endocardial fibroelastosis in congenital pulmonic stenosis highlighting the utility of the FIDDLE sequence. Finally, the prestigious SCMR Gold medal awards for 2022, the highest honour bestowed by the Society, went to two stalwarts who both have made huge contributions to the field of CMR—Dr. James Moon, Professor of Cardiology at University College London, England and CMR director at Barts Heart Centre, and Dr. Robert Edelman, Chairman of Radiology, Northshore Research Institute, Evanston, Illinois, USA.

**Fig. 3:**
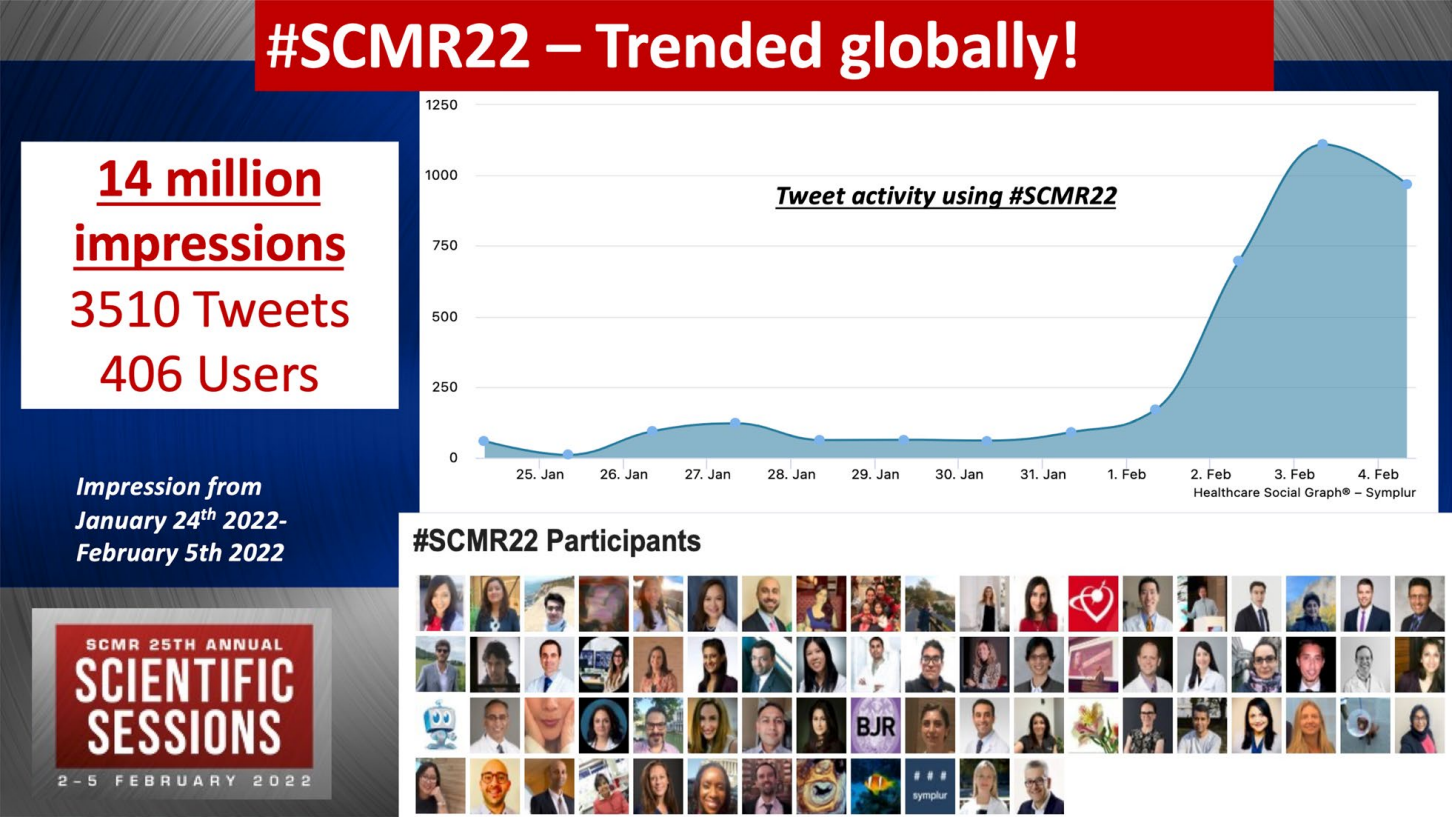
The social media statistics for #SCMR22. 14 million impressions were created during January 24–February 5, 2022 (Data from symplur.com)

As always social media and advocacy played a great role in disseminating science across the world. As highlighted by Dr. Michael Salerno in the closing ceremony, the #SCMR22 trended globally during the meeting with over 14 million impressions created worldwide through January 24–February 5, 2022, with 3510 tweets and more than 405 users (Fig. 3). The best tweet award for the most educational tweet during the conference was awarded to Dr. Anudeep Dodeja for covering parametric mapping in myocarditis and cardiomyopathy. Dr. Amro Alsaid and Dr. Jennifer Co-Vu were the runners up for this contest.

## Vision for the future: shaping the future of CMR

The 25th SCMR Annual Scientific Sessions was a unique blend of state-of-the-art lectures covering both clinical and translational components of CMR, demonstration of various innovations, interesting cases and debates covering almost all the aspects of current day CMR practice. CMR is poised for the paradigm shift towards providing value, with increased recognition in clinical practice guidelines. The path into the future will be to utilize newer computational and quantitative techniques to further improve the value of CMR by enabling more efficient and simplified acquisition, and to improve CMR outreach world-wide towards the ultimate goals of making CMR accessible anywhere. As Prof Jeanette Schulz-Menger rightly said in her opening plenary lecture, CMR is “an enabler of precision medicine”. Moving towards the widespread adoption of CMR, the SCMR will align itself with CMR communities in various countries as well as other imaging societies to enable a broader dissemination of CMR into clinical practice worldwide.

With the rapid pace of developments, we expect to see more transformative science in the coming years. The major areas of focus are likely to be rapid efficient and high value CMR exams, automated and quantitative acquisition and post-processing using AI and machine learning, multi-contrast imaging and advanced vascular imaging including 4D flow. In the coming years, we look forward to more transformative scientific content at the SCMR scientific sessions aligned with the theme “Changing Global Clinical Practice”. Indeed, the increasing participation of countries outside of the United States and Europe as well as outreach efforts of the SCMR in developing countries are testaments to SCMR’s global mission. It is an exciting and pivotal time for CMR. We look forward to welcoming all of you to the 2023 SCMR Annual Scientific Sessions from January 25–28, 2023 in sunny San Diego, California, USA.

## Data Availability

Review article, not applicable.

## Abbreviations

ACC: American College of Cardiology
AI: Artificial intelligence
AS: Aortic stenosis
CAD: Coronary artery disease
CHD: Congenital heart disease
CMR: Cardiovascular magnetic resonance
CNN: Convolutional neural networks
COVID-19: Coronavirus disease 2019
CT: Computed tomography
ECV: Extracellular volume
INOCA: Ischemia and no obstructive coronary artery disease
ISMRM: International Society of Magnetic Resonance in Medicine
LGE: Late gadolinium enhancement
LV: Left ventricle/left ventricular
MBF: Myocardial blood flow
MINOCA: Myocardial infarction with no obstructive coronary arteries
MPR: Myocardial perfusion reserve
MVD: Microvascular dysfunction
NICM: Non-ischemic cardiomyopathy
PET: Positron emission tomography
RV: Right ventricle/right ventricular
SCMR: Society for Cardiovascular Magnetic Resonance
VHD: Valvular heart disease
